# Role of Serum Vaspin in Progression of Type 2 Diabetes: A 2-Year Cohort Study

**DOI:** 10.1371/journal.pone.0094763

**Published:** 2014-04-14

**Authors:** Weixia Jian, Wenhui Peng, Sumei Xiao, Hailing Li, Jie Jin, Li Qin, Yan Dong, Qing Su

**Affiliations:** 1 Department of Endocrinology, Xinhua Hospital, Shanghai Jiaotong University School of Medicine, Shanghai, China; 2 Department of Cardiology, Shanghai Tenth People's Hospital, Tongji University School of Medicine, Shanghai, China; 3 Department of Medicine, Faculty of Medicine, The University of Hong Kong, Pokfulam, Hong Kong; University of Lille Nord de France, France

## Abstract

Vaspin is a novel adipocytokine that has potential insulin-sensitizing effects. The aim of this study is to explore the role of vaspin in the progression of type 2 diabetes mellitus (T2DM) in humans through a longitudinal process. This was a 2-year follow-up study that included 132 patients with T2DM and 170 non-diabetic subjects. The serum vaspin and adiponectin levels were determined with ELISA. Anthropometric measurements, circulating glucose, hemoglobin A1c, insulin level, liver function, kidney function, and lipid profile were measured for each participant. The new onset of T2DM was counted in non-diabetic subjects and the glycemic control was analyzed in T2DM patients at follow-up. At enrollment, the serum vaspin and adiponectin levels were lower in T2DM patients compared with non-diabetic subjects. Significant positive correlation between serum vaspin and HDL-C levels (r = 0.23, *P* = 0.006) was observed in non-diabetic controls. The serum vaspin concentration was also significantly correlated with body mass index (BMI) (r = 0.19, *P* = 0.028), waist-hip ratio (WHR) (r = 0.17, *P* = 0.035) and homeostasis model assessment of insulin resistance (HOMA-IR) (r = 0.14, *P* = 0.029) in T2DM patients. In cohort analyses, it was found that lower serum vaspin [odds ratio (OR) = 0.52, 95% confidence interval (CI): 0.10–0.87, *P* = 0.015] and adiponectin (OR = 0.35, 95% CI: 0.20–0.72, *P* = 0.015) levels at baseline were risk factors for new onset of T2DM at follow-up. The percentage of insulin treatment in T2DM patients was higher in the sub-group with lower serum vaspin level than that in the sub-group with higher vaspin level at follow-up (55.3% vs. 44.7%, *P* = 0.020). Our study indicates that low serum concentration of vaspin is a risk factor for the progression of T2DM.

## Introduction

Adipose tissue is not only an inert energy-storing tissue, but also an active endocrine organ [1], which secrets many kinds of adipocytokines, such as adiponectin, leptin, TNF-α and so on. Some adipocytokines are involved in the development of insulin resistance, which is an important pathological link among various metabolic dysfunctions, including obesity, diabetes and cardiovascular diseases [1]. Among the adipocytokines, adiponectin is one of the most-studied and proved to have beneficial effects on insulin resistance. Adiponectin ameliorates insulin sensitivity and stimulates fatty acid oxidation in skeletal muscle and also has anti-inflammatory effects in blood vessels [2]–[6]. Lower serum concentration of adiponectin also predicts higher risk of diabetes in human, suggesting an important role of adiponectin that links obesity, insulin resistance, and type 2 diabetes mellitus (T2DM) [7]–[9].

Vaspin (visceral adipose tissue-derived serine protease inhibitor), a novel adipocytokine, was firstly identified in obese OLETF rats. It has been suggested that vaspin has potential insulin-sensitizing effects [10]. Vaspin significantly improves glucose tolerance and insulin sensitivity in diet-induced obese mice. In db/db mice, vaspin treatment is associated with sustained glucose-lowering effects for at least 6 days after the injection [11]. In humans, vaspin expression in terms of mRNA was detected in human visceral and subcutaneous adipose tissue [12]. Recent studies also found that vaspin gene expression in human adipose tissue and circulating vaspin levels were positively associated with obesity-associated diseases and T2DM [13]–[16]. Furthermore, it is indicated that vaspin plays a role in adipoinsular axis, and may be associated with insulin resistance in obese subjects, including patients with T2DM and polycystic ovary syndrome [13]. Therefore, all these data suggest that vaspin may be involved in the glucose metabolism and the development of T2DM in human. Vaspin has been shown to significantly improve glucose tolerance and insulin sensitivity in murine and to be positively associated with obesity-related diseases in human [14], which seems to be conflicting with each other. Hida K's study demonstrated that vaspin was barely detectable in OLETF rat at 6 weeks and was highly expressed in adipocytes of visceral white adipose tissues at 30 weeks, the age when obesity, body weight, and insulin levels peak in OLETF rats. The tissue expression of vaspin and its serum levels decreased with worsening of diabetes and body weight loss at 50 wk [10]. These results indicate that the levels of serum vaspin may change with the progression of diabetes. Vaspin may increase at the beginning and decrease with worsening of diabetes in human. A significant correlation between serum vaspin and leptin concentrations supports previous human studies that serum vaspin concentration reflects body fat mass in human [17]. Vaspin may have a compensatory role in insulin resistance in human obesity-associated diseases. Up to date, all studies on roles of vaspin in human metabolic diseases were cross-sectional, but not cohort studies. Therefore, it is still unclear what the real role of vaspin is in the progression of diabetes in a longitudinal process.

Hypothetically, just like adiponectin, low vaspin level might be concomitant with poor glycemic control in diabetic patients because of its impact on insulin sensitivity and glucose metabolism. To our knowledge, the role of vaspin in glycemic control in diabetic patients has not been addressed in previous studies. In this 2.0-year follow-up study, our aims were to examine 1) whether serum vaspin levels are associated with adiposity and insulin-resistance; 2) whether non-diabetic subjects with lower serum vaspin levels are more susceptible to developing T2DM; 3) whether lower vaspin levels are concomitant with poorer glycemic control in T2DM.

## Materials and Methods

### Subjects

All subjects were local residents of Han ethnicity in Shanghai and consecutively recruited in Department of Endocrinology, Xinhua hospital, Shanghai Jiaotong University School of Medicine and Department of Cardiology, Shanghai Tenth People's Hospital, Tongji University School of Medicine from Oct 2009 to Mar 2010. T2DM was diagnosed according to 1999 WHO diagnostic criteria [18]. The non-diabetic subjects were recruited from those in-patients admitted to hospital due to hypertension or other mild cardiovascular symptoms. Subjects with mild cardiovascular symptoms were those with chief complaints including mild chest distress or chest pain, excluding acute coronary syndrome or acute myocardial infarction by simultaneous coronary angiography, electrocardiogram, and tests of myocardial enzymes. The diagnosis of type 1 diabetes (T1DM) was confirmed by c peptide level and presence of glutamic acid decarboxylase antibody. Patients with T1DM, severe kidney or liver diseases, severe cardiovascular diseases, chronic viral or bacterial infection, asthma, tumors, and connective tissue diseases were excluded. Non-diabetic subjects in our study were those simultaneously match the following three criteria: fasting plasma glucose (FPG) concentration less than 6.1 mmol/L; 2-hour postprandial plasma glucose (2h-PG) concentration less than 7.8 mmmol/L and glycosylated haemoglobin A1c (HbA1c) less than 6.0%. The clinical information and anthropometric indices for all subjects at baseline and follow-up were obtained by standard methods.

The study was approved by the Human Ethical Review Committees, Xinhua Hospital and Shanghai Tenth People's Hospital, and performed in accordance with the Declaration of Helsinki. Written informed consent was obtained from all subjects.

### Biochemical Assays

Blood samples for biochemical analysis were collected after overnight fasting for at least 10 hours, and immediately frozen in aliquots at −80°C until analysis. FPG, 2h-PG, serum liver and renal function and lipid profile were measured by using standard laboratory techniques on a Hitachi 7104 Analyzer (Hitachi, Tokyo, Japan). HbA1c was determined by using high-performance liquid chromatography (Hi-AUTO HA-8150, ARKRAY, Kyoto, Japan) method. Serum insulin concentrations were determined by commercially available radioimmunoassay in non-diabetic subjects and insulin-naive T2DM patients (Linco Res., St. Charles, MO, USA). Insulin sensitivity index: homeostasis model assessment of insulin resistance (HOMA-IR) was calculated by the formula as reported before [19]. Serum vaspin and adiponectin levels were determined in all subjects at baseline. Serum vaspin (Adipogen, Seoul, South Korea) and total adiponectin (R&D Systems, Minneapolis, MN, USA) levels were analyzed by using commercially available ELISAs as indicated before [20]. The degree of precision of the ELISA system in terms of coefficient of variance (percent) of intra-assay was between 1.31% and 1.74%, and that of inter-assays was between 5.9% and 8.3%. Because Teshigawara S's study in Japanese population demonstrated that about 7% of higher vaspin levels more than 10 ng/ml was beyond the detection range of vaspin ELISA kit from Adipogen company [21], three subjects with vaspin levels more than 10 ng/ml in our sample were excluded.

### Follow-up

At re-examination after two years, anthropometric indices, FPG, 2h-PG, HbA1c and lipid profile were measured in all subjects. Drug uses and concomitant diseases were also recorded at the same time. During the 2-year follow-up, FPG and HbA1c had been checked in diabetic patients every three months at clinic, and hypoglycemic agents were prescribed by experienced endocrinologists. For diabetic patients, during the stable therapeutical period of two years, insulin or oral antidiabetics (OAD) treatments were recorded. For non-diabetic controls, the incidence of T2DM was investigated, and the glucose levels and other metabolic indices were also analyzed at follow-up.

### Statistical analysis

Statistical analysis was performed using the Statistical Package for the Social Sciences (SPSS v.11.0, SPSS Inc., Chicago, IL, USA). All datasets were examined for normal distribution using the Kolmogorov-Smirnov test. The traits that deviated significantly from normal distribution were approximately normalized by logarithmic transformation. Differences between groups were examined using an unpaired *t* test or covariance analysis for continuous variables and a χ^2^ test for categorical variables. Pearson correlation coefficients were used to assess the associations of continuous variables with vaspin. Multiple stepwise logistic regression analysis was used to detect the risk factors for development of T2DM, with adjustment for potential confounding factors. Results are presented as mean (standard deviation) for continuous variables and as percentage for categorical variables. The serum vaspin and adiponectin concentrations were adjusted for sex in all analyses because of the higher levels of these hormones in women [22], [23]. The baseline vaspin and adiponectin concentrations of the entire cohort were grouped into two sub-groups in a sex-specific manner so that each subject was classified as being under or above the mean of vaspin and adiponectin level among individuals of the same sex. A 2-sided probability level of P≤0.05 was taken as significant.

## Results

### Clinical and biochemical characteristics of subjects at baseline

A total of 148 T2DM patients and 193 non-diabetic controls were included in the baseline analysis. Decreased vaspin levels were found in T2DM group compared with non-diabetic control group (*P* = 0.025). The *P* value was 0.041 after adjusting for the confounding factors including gender, age and BMI. The median (interquartile range) level of vaspin was 0.425 ng/mL (0.160, 0.917 ng/mL) in non-diabetic group, and 0.353 ng/mL (0.191, 0.664 ng/mL) in T2DM group. Higher adiponectin [median (interquartile range): 10.525 µg/ml (5.977, 15.550) vs. 7.310 µg/ml (4.034, 13.113), *P* = 0.05] was found in non-diabetic controls when compared with T2DM group. The *P* value was not significant after adjusting for the confounding factors including gender, age and BMI. The serum fasting insulin level was significantly higher in T2DM group than that in control group (*P*<0.05). In females with or without diabetes, both the serum vaspin and adiponectin levels were significantly higher than those in males (all *P*<0.05). Detailed baseline clinical characteristics of all subjects were presented in Table S1. In control group, compared with subjects with lower vaspin levels, those with higher vaspin levels had significantly higher WHR (0.87±0.05 vs. 0.80±0.02, *P* = 0.025) and lower percentage of smokers (7.2% vs. 22.0% *P* = 0.003). In T2DM group, significantly higher HDL-C (1.2±0.4 vs. 1.1±0.3, *P* = 0.043) and HOMA-IR (2.49±1.76 vs. 1.86±0.90, *P* = 0.004) were found in sub-group with higher vaspin levels (Table 1).

**Table 1:** Basic clinical characteristics of all subjects at baseline.

	Control group (193)			T2DM group (148)		
	Low vaspin (82)	High vaspin (111)	*P* value	Low vaspin (66)	High vaspin (82)	*P* value
Age (years)	62±12	59±12	0.287	62±12	64±10	0.218
Male, n (%)	58 (70.7%)	50 (45.0%)	<0.001	41 (62.1%)	26 (39.4%)	0.009
Smoking, n (%)	18 (22.0%)	8 (7.2%)	0.003	23 (34.8%)	27 (24.2%)	0.182
Hypertension, n (%)	40 (48.8)	49 (44.1%)	0.523	28 (42.4%)	39 (47.6%)	0.533
Obesity, n (%)	13 (15.9%)	15 (13.5%)	0.648	11(16.7%)	19(23.2%)	0.328
Dislipidemia, n (%)	12 (14.6%)	13 (11.7%)	0.550	13 (19.7%)	18 (22.0%)	0.738
Duration of T2DM	-	-		9.2±7.4	8.8±7.4	0.654
BMI (kg/m^2^)	24.6±3.4	23.7±3.5	0.320	25.0±4.1	27.0±5.2	0.054
WHR	0.80±0.02	0.87±0.05	0.025	0.92±0.07	0.93±0.08	0.593
SBP (mmHg)	143±25	138±20	0.056	135±18	141±22	0.059
DBP (mmHg)	82±13	81±13	0.333	79±11	81±11	0.281
BUN (mmol/L)	5.79±1.75	6.24±3.55	0.310	5.9±2.1	6.3±2.8	0.348
Creatinine (µmol/L)	68.3±22.0	70.9±44.7	0.508	68.4±23.6	66.4±26.4	0.640
TC (mmol/L)	4.5±1.1	4.8±0.9	0.090	4.6±1.0	4.8±1.1	0.375
TG (mmol/L)	1.6±1.5	1.5±0.6	0.189	1.9±1.6	2.5±4.0	0.269
HDL-C (mmol/L)	1.1±0.3	1.2±0.3	0.580	1.1±0.3	1.2±0.4	0.043
LDL-C (mmol/L)	2.6±0.8	2.8±0.7	0.331	2.7±0.8	2.7±0.8	0.661
FPG (mmol/L)	5.2±0.9	5.1±0.6	0.660	8.1±3.1	7.9±2.6	0.580
2h-PG (mmol/L)	6.6±3.0	6.9±1.9	0.659	13.5±4.8	13.7±4.5	0.791
HbA1c (%)	5.4±1.2	5.5±1.1	0.872	8.7±2.1	8.7±2.0	0.953
Fasting insulin (mIU/L)	7.7±5.1	7.8±4.1	0.960	8.8±9.2	11.5±14.2	0.287
HOMA-IR*	1.90±1.30	1.81±0.95	0.812	1.86±0.90	2.49±1.76	0.004
Vaspin (ng/mL)*	0.15±0.09	2.35±3.30		0.19±0.09	0.81±0.54	
Adiponectin (µg/ml)*	12.57±10.25	12.25±10.31	0.819	10.10±6.71	7.68±4.59	0.187

BMI: body mass index; WHR: waist-hip ratio; SBP: systolic blood pressure; DBP: diastolic blood pressure; BUN: blood urea nitrogen; TC: total cholesterol; TG: triglycerides; HDL-C: high-density lipoprotein cholesterol; LDL-C: low density lipoprotein-cholesterol; FPG: fasting plasma glucose; 2h-PG: 2-h postprandial plasma glucose; HbA1c: glycosylated haemoglobin A1c; HOMA-IR: homeostasis model assessment of insulin resistance;

*logarithmically transformed before analysis.

### Correlations between vaspin and anthropometric and biochemical indices

Significant positive correlation between vaspin and HDL-C (r = 0.23, *P* = 0.006) was found in controls. In T2DM group, vaspin was significantly positively correlated with BMI (r = 0.19, *P* = 0.028), WHR (r = 0.17, *P* = 0.035) and HOMA-IR (r = 0.14, *P* = 0.029). All results of Pearson correlation analysis were shown in Table 2. In either control or T2DM group, vaspin level did not correlate with glycemic measurements including FPG, 2h-PG and HbA1c. Vaspin level did not correlate with adiponectin level in either group.

**Table 2:** Results of Pearson correlations of vaspin with clinical and biomedical indices at baseline.

	Control group		T2DM group	
	r value	*P* value	r value	*P* value
Age	−0.01	0.876	0.10	0.263
Duration of T2DM	-	-	0.03	0.709
BMI	−0.10	0.298	0.19	0.028
WHR	0.21	0.682	0.17	0.035
TC	0.06	0.451	−0.01	0.924
TG	−0.03	0.711	0.10	0.242
LDL-C	−0.04	0.599	−0.13	0.126
HDL-C	0.23	0.006	0.15	0.090
FPG (mmol/L)	−0.04	0.589	0.03	0.688
2h-PG (mmol/L)	−0.03	0.857	0.07	0.431
HbA1c (%)	0.05	0.733	0.01	0.940
Fasting insulin	0.11	0.460	0.01	0.930
HOMA-IR^*^	0.03	0.843	0.14	0.029
adiponectin	0.13	0.101	−0.13	0.420

BMI: body mass index; WHR: waist-hip ratio; TC: total cholesterol; TG: triglycerides; HDL-C: high-density lipoprotein cholesterol; LDL-C: low density lipoprotein-cholesterol; FPG: fasting plasma glucose; 2h-PG: 2-h postprandial plasma glucose; HbA1c: glycosylated haemoglobin A1c; HOMA-IR: homeostasis model assessment of insulin resistance;

*logarithmically transformed before analysis.

### Results of the follow-up study

In this study, 170 non-diabetic subjects and 132 T2DM patients were followed up for 2 years. Compared with baseline characteristics, T2DM patients had significantly lower HbA1c, higher HDL and higher percentage of insulin treatment at follow-up (data not shown).

### 1) Occurrence of T2DM in control group

**Figure 1:**
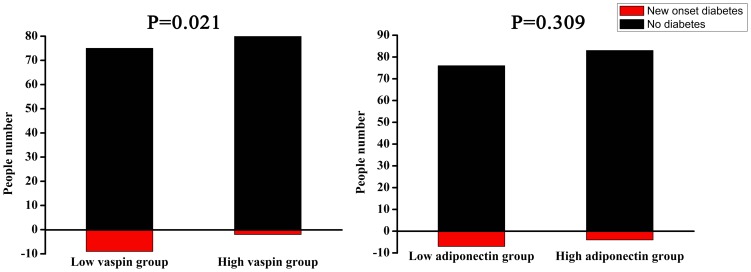
Percentages of new occurrences of T2DM in control subjects with low or high vaspin and adiponectin levels at follow-up. Red and black bars represent new onset diabetic patients and non-diabetic subjects in control group at follow-up, respectively. χ^2^ test was used to analyze the difference of percentage of new onset diabetic patients in sub-groups with low and high vaspin levels.

Among 170 subjects without T2DM at baseline, 11 developed T2DM during follow-up. 3 and 6 subjects were diagnosed with impaired glucose tolerance (IGT) and impaired fasting glucose (IFG) respectively. As were shown in Figure 1, higher percentage of new onset of T2DM was found in sub-group with low vaspin levels than in sub-group with high vaspin levels (11.0% vs. 2.3%, *P* = 0.021). Similarly, higher percentage of new onset of T2DM was also found in sub-group with low adiponectin levels, compared with sub-group with high adiponectin levels. However, the statistical analysis was not significant (8.4% vs. 4.6%, *P* = 0.309). It was found that higher FPG and BMI, lower serum vaspin and adiponectin levels at baseline were independent risk factors for the occurrence of T2DM during 2.0-year follow-up. Results of multiple stepwise logistic regression analysis were shown in Table 3, and the values of odds ratio of vaspin and adiponectin were 0.52 (95% CI: 0.10–0.87, *P* = 0.015) and 0.35 (95% CI: 0.20–0.72, *P* = 0.015), respectively.

**Table 3:** Results of multiple stepwise logistic regression analysis for risk factors for new onset of T2DM in control group at follow-up.

	OR value	95% CI	*P* value
FPG	9.81	1.80–55.44	0.009
BMI	1.03	1.01–1.29	0.050
Adiponectin	0.35	0.20–0.72	0.015
Vaspin	0.52	0.10–0.87	0.015

FPG: fasting plasma glucose; BMI: body mass index.

### 2) Glucose levels in control and T2DM groups

**Figure 2:**
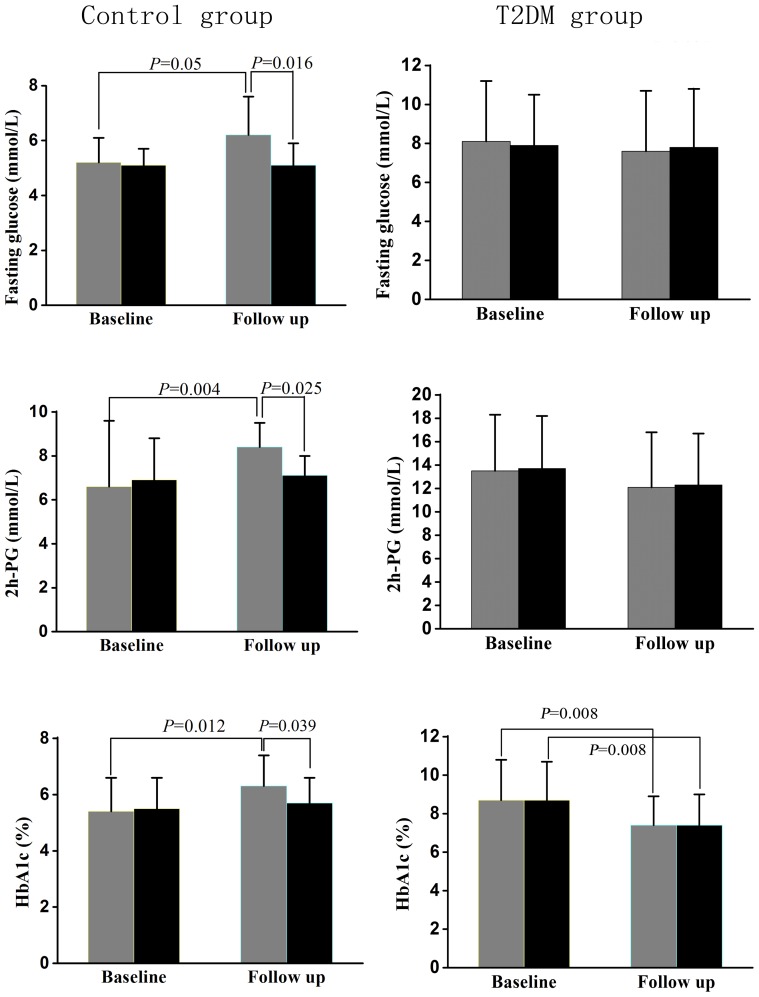
Glucose levels in groups with low and high serum vaspin concentrations. Gray and black bars represent diabetic patients with low and high vaspin levels, respectively. Fasting plasma glucose (FPG), 2-hour postprandial plasma glucose (2h-PG) and glycosylated haemoglobin A1c (HbA1c) were compared between those at baseline and follow-up in control and T2DM groups. Covariance analysis was used to analyze the differences between sub-groups.

For sub-group with low vaspin levels in control group, significantly increased FPG, 2h-PG and HbA1c at follow-up were found when compared with those at baseline (*P*≤0.05). At follow-up, FPG (5.1±0.8 vs. 6.4±1.4, *P* = 0.016), 2h-PG (7.2±0.9 vs. 8.4±1.2, *P* = 0.025) and HbA1c (5.6±0.8 vs. 6.3±1.2, *P* = 0.039) were all significantly lower in sub-group with high vaspin levels than those in sub-group with low vaspin levels. In T2DM group, HbA1c was significantly lowered in sub-groups with either low or high vaspin levels at follow-up than baseline (both *P* = 0.008). There was no significant difference of the glycemic control between sub-groups with low and high vaspin levels at follow-up. (Results were shown in Figure 2).

### 3) Insulin treatment in T2DM group

As were shown in Table 4, among 132 T2DM patients, 67 patients were administrated with insulin at baseline and 27 additional patients who were insulin-native at baseline had been administrated with insulin during follow-up. At baseline, there was no significant difference of percentages of insulin-treated patients between sub-groups with low and high vaspin levels. At follow-up, higher percentage of insulin treatment in T2DM patients was found in sub-group with low vaspin level than sub-group with high vaspin level (55.3% vs. 44.7%, *P* = 0.020). Compared with sub-group with low adiponectin level, sub-group with high adiponectin level had less patients receiving insulin treatment both at baseline (37.3% vs. 62.7%, *P* = 0.009) and follow-up (42.6% vs. 57.4%, *P* = 0.027).

**Table 4:** Medicine treatment in T2DM group with low or high vaspin and adiponectin levels at baseline and follow-up.

	Low vaspin	High vaspin	*P* value	Low adiponectin	High adiponectin	*P* value
Baseline hypoglycemic therapy						
One oral hypoglycemic agent	15 (41.7%)	21 (58.3%)		15 (40.5%)	21 (59.5%)	
Two or more oral hypoglycemic agents	16 (55.2%)	13 (44.8%)		9 (45.0%)	20 (55.0%)	
Insulin treatment	35 (52.2%)	32 (47.8%)	0.486	42 (62.7%)	25 (37.3%)	0.009
Follow-up						
One oral hypoglycemic agent	5 (22.7%)	17 (77.3%)		7 (31.8%)	15 (68.2%)	
Two or more oral hypoglycemic agents	9 (52.3%)	7 (47.7%)		5 (45.5%)	11 (54.5%)	0.027
Insulin treatment	52 (55.3%)	42 (44.7%)	0.020	54 (57.4%)	40 (42.6%)	

## Discussion

Vaspin is a novel adipocytokine that is supposed to have insulin sensitizing effects [10]. However, the real role of vaspin on glucose dysregulation in humans is not well understood. Our longitudinal study analyzed the independent roles of vaspin and adiponectin in the incidence of T2DM in non-diabetic subjects and long-time glycemic control in patients with T2DM. We herein present novel evidence that decreased baseline serum vaspin is an independent risk factor for subsequent occurrence of diabetes in non-diabetic subjects and higher percentage of insulin treatment in diabetic patients.

Although results of animal studies indicate that vaspin is an insulin-sensitizing adipocytokine, the real role of vaspin in human insulin resistance is still unclear [23], [24]. A study performed in adolescents found that the serum vaspin concentration increased with worsening insulin resistance and was acutely down-regulated following glucose provocation in insulin-resistant adolescents [25]. In overweight women with polycystic ovarian syndrome, vaspin was found to be positively associated with BMI and WHR. Furthermore, the expression of vaspin in omental adipose tissue was reduced by metformin treatment in accordance with the decrease in insulin resistance [13]. In another study performed in the morbidly obese women, the vaspin mRNA expression was significantly higher in both subcutaneous and visceral adipose tissue than lean controls [26]. However, inconsistent results were also found in other studies. In Youn's study, no difference was found in circulating vaspin levels between individuals with normal glucose tolerance and T2DM. T2DM seems to abrogate the correlation between circulating vaspin, BMI, and insulin sensitivity [24]. In another cross-sectional study performed in normal volunteers, glucose tolerance status and insulin sensitivity measured using euglycemic hyperinsulinemic clamp and HOMA-IR were not found to be associated with serum vaspin levels [27]. In our study, obese diabetic patients had a tendency of having increased serum vaspin, and increased HOMA-IR was found in diabetic patients with higher vaspin levels. In the correlation analysis, we have observed a significant positive association of vaspin with HOMA-IR in diabetic group and HDL-C in control group, which was consistent with the results from some other studies [23], [28]. The latter studies found that circulating vaspin level was positively correlated with HDL-C in healthy subjects and negatively correlated with detrimental lipid parameters (TC, TG and LDL-C) in pregnant women. Results from the present and previous studies suggest that vaspin plays a role in human insulin resistance and lipid metabolism [29]. Vaspin may be a compensatory molecule in the pathogenesis of insulin resistance and obesity associated diseases [30]. It can be postulated that vaspin, as a serine protease inhibitor, inhibits a protease which plays a role in the degradation of a hormone or molecule with direct or indirect glucose and lipid lowering effects [11], [31]. In our study, lower vaspin levels were found in T2DM patients than those in control subjects. This result may be due to the long mean diabetes duration of our T2DM patients (10.5 years). The long diabetes duration and insulin treatment may affect the vaspin levels and thus induce the decrease of vaspin in our T2DM group than in non-diabetic group [10], [23].

Gulcelik's research found that serum vaspin levels in diabetic patients with chronic complications including neuropathy, retinopathy and nephropathy were lower than those without above complications [32]. Another study detected lower serum concentrations of vaspin in patients with carotid stenosis who experienced an ischemic event compared with asymptomatic patients [17]. The above results indicate that diabetic patients with lower vaspin levels may have poor outcomes and higher prevalence of micro- or macro-vascular complications during a long time. In our T2DM patients at follow-up, there were no significant associations between serum vaspin levels and glycemic controls. This result may be due to the confounding effects of drug administration during the follow-up. However, higher percentage of insulin administration at follow-up in diabetic patients was found in those with lower vaspin levels. In non-diabetic group, it was also found that lower vaspin level was associated with elevated glycemic levels and higher incidence of T2DM at follow-up. From above results, it could be speculated that low vaspin levels might be involved in poor glycemic control and other detrimental metabolic effects, and thus play a role in development of chronic diabetic complications in T2DM patients.

In our study, it was found that adiponectin was an independent risk factor for new onset of T2DM in non-diabetic subjects at follow-up, which was accordant with some other studies [33], [34]. Even though adiponectin and vaspin may both play beneficial roles in insulin resistance, the reported relationships between serum vaspin and adiponectin were controversial. In Giomisi's investigation, significant positive association between serum vaspin and adiponectin levels was detected in women of child-bearing age [28]. However, in a study performed in pubertal obese children and adolescents, vaspin levels were negatively correlated with adiponectin levels [35]. In another study performed in mice, it was found that increased expression of adiponectin was detected in high fat high sucrose chow ICR mice treated with vaspin [10]. In the correlation analysis of our study, the serum vaspin levels did not correlate with serum adiponectin levels. These results indicate that the relationship between serum adiponectin and vaspin levels can be affected by age, body weight and other metabolic status.

Several limitations should be considered before results interpretation. First, the control subjects were not normal people, but inpatients with hypertension or other mild cardiovascular diseases. This inclusion method can not exclude some confounding effects, including the effects of diseases itself and drugs treatment. However, people with high risk of T2DM constituted the control group, which might be partially responsible for the high occurrence of T2DM at follow-up and higher statistical power in small sample size. Second, the T2DM patients were not enrolled at the onset of the disease. The different duration of T2DM and different treatment in each patient may incorporate a possible source of selection bias. Third, the relatively small sample size in this study will decrease the statistical power, and further studies with larger sample size should be performed in the future to validate our results. Fourth, the relatively short follow-up period will weaken the effects of vaspin on glucose levels and chronic complications in T2DM group. Nevertheless, this cohort study provides support to the role of vaspin in glycemic control in non-diabetic people.

In conclusion, low circulating vaspin level, as well as adiponectin level can be used as risk factors for the progression of T2DM. Further prospective observational studies are required to explain how a decrease in circulating vaspin level may be involved in the progression of T2DM.

## Supporting Information

Table S1 **Basic clinical characteristics of all subjects at baseline.** Note: BMI: body mass index; WHR: waist-hip ratio; SBP: systolic blood pressure; DBP: diastolic blood pressure; BUN: blood urea nitrogen; TC: total cholesterol; TG: triglycerides; HDL-C: high-density lipoprotein cholesterol; LDL-C: low density lipoprotein-cholesterol; FPG: fasting plasma glucose; 2h-PG: 2-h postprandial plasma glucose; HbA1c: glycosylated haemoglobin A1c; HOMA-IR: homeostasis model assessment of insulin resistance;*logarithmically transformed before analysis. Adjusted P values were from covariance analysis by adjusting for age, sex and BMI.
(DOC)
