# Supplementary material for: Role of Serum Vaspin in Progression of Type 2 Diabetes: A 2-Year Cohort Study
Source: PLoS One. 2014 Apr 14;9(4):e94763. doi: 10.1371/journal.pone.0094763 (PMC3986225; doi:10.1371/journal.pone.0094763)
Supplement: Table S1 — Basic clinical characteristics of all subjects at baseline. Note: BMI: body mass index; WHR: waist-hip ratio; SBP: systolic blood pressure; DBP: diastolic blood pressure; BUN: blood urea nitrogen; TC: total cholesterol; TG: triglycerides; HDL-C: high-density lipoprotein cholesterol; LDL-C: low density lipoprotein-cholesterol; FPG: fasting plasma glucose; 2h-PG: 2-h postprandial plasma glucose; HbA1c: glycosylated haemoglobin A1c; HOMA-IR: homeostasis model assessment of insulin resistance;*logarithmically transformed before analysis. Adjusted P values were from covariance analysis by adjusting for age, sex and BMI. (DOC) [file pone.0094763.s001.doc]

Table S1 Basic clinical characteristics of all subjects at baseline

|  | Non-diabetic controls (193) | T2DM patients (148) | *P* values | Adjusted *P* values |
| --- | --- | --- | --- | --- |
| Age (years) | 60.3±12.2 | 63.1±11.3 | 0.075 | - |
| Male, n (%) | 108 (56.0%) | 67 (45.3%) | 0.050 | - |
| Smoking, n (%) | 26 (13.5%) | 50 (33.8%) | <0.001 | - |
| BMI (kg/m^2^) | 24.1±3.5 | 26.1±4.3 | <0.001 | - |
| WHR | 0.84±0.04 | 0.93±0.08 | <0.001 | - |
| SBP (mmHg) | 139.9±23.6 | 138.3±20.2 | 0.875 | - |
| DBP (mmHg) | 81.4±13.3 | 80.1±11.2 | 0.743 | - |
| BUN (mmol/L) | 6.05±2.59 | 6.12±2.42 | 0.269 | 0.295 |
| Creatinine (μmol/L) | 69.79±32.49 | 67.29±24.36 | 0.315 | 0.399 |
| TC (mmol/L) | 4.69±1.05 | 4.71±1.14 | 0.677 | 0.756 |
| TG (mmol/L) | 1.53±0.78 | 2.23±2.94 | 0.001 | 0.001 |
| HDL-C (mmol/L) | 1.15±0.30 | 1.16±0.33 | 0.757 | 0.769 |
| LDL-C (mmol/L) | 2.69±0.73 | 2.69±0.77 | 0.302 | 0.804 |
| FPG (mmol/L) | 5.17±0.78 | 7.99±2.81 | <0.001 | <0.001 |
| 2h-PG (mmol/L) | 6.75±2.69 | 13.61±4.57 | <0.001 | <0.001 |
| HbA1C (%) | 5.45±1.08 | 8.69±2.27 | <0.001 | <0.001 |
| Fasting insulin (mIU/L) | 7.77±5.08 | 10.30±4.97 | 0.020 | 0.043 |
| HOMA-IR^＊^ | 1.85±1.09 | 2.21±1.49 | 0.315 | 0.456 |
| Vaspin (ng/mL) ^＊^ | 0.425 (0.160, 0.917) | 0.353 (0.191, 0.664) | 0.025 | 0.041 |
| Adiponectin (μg/ml) ^＊^ | 10.525 (5.977, 15.550) | 7.310 (4.034, 13.113) | 0.050 | 0.076 |

BMI: body mass index; WHR: waist-hip ratio; SBP: systolic blood pressure; DBP: diastolic blood pressure; BUN: blood urea nitrogen; TC: total cholesterol; TG: triglycerides; HDL-C: high-density lipoprotein cholesterol; LDL-C: low density lipoprotein-cholesterol; FPG: fasting plasma glucose; 2h-PG: 2-h postprandial plasma glucose; HbA1c: glycosylated haemoglobin A1c; HOMA-IR: homeostasis model assessment of insulin resistance;＊logarithmically transformed before analysis. Adjusted *P* values were from covariance analysis by adjusting for age, sex and BMI.
